# Finerenone: a breakthrough mineralocorticoid receptor antagonist for heart failure, diabetes and chronic kidney disease

**DOI:** 10.1186/s43044-024-00586-z

**Published:** 2024-12-16

**Authors:** Akshyaya Pradhan, Umesh Chandra Tripathi

**Affiliations:** 1 Department of Cardiology, King George’s Medical University, Lucknow, Uttar Pradesh 226003 India; 2 Department of Cardiology, Sanjay Gandhi Post Graduate Institute of Medical Sciences, Lucknow, Uttar Pradesh 226014 India

## Abstract

**Background:**

Aldosterone is categorized as a mineralocorticoid hormone produced in the zona glomerulosa of the adrenal cortex. Aldosterone has considerable action in sodium and water retention along with cardiac remodeling, promoting fibrosis and these detrimental effects have been counteracted by mineralocorticoid receptors antagonists over time. Spironolactone, a non-selective steroidal MRA used extensively is potent but has serious adverse effects like gynecomastia and hyperkalemia. Eplerenone another second generation MRA, though non-steroidal and selective causes hyperkalemia and adversely effecting renal functions.

**Main body:**

Recently Finerenone- a novel MRA has been introduced which is as potent like spironolactone with less adverse effects and improved cardiovascular outcomes particularly in chronic kidney failure with diabetes. The article reviews the physical and chemical properties of Finerenone and compares it with MRAs already in use, and then about the patient specific uses of Finerenone and future avenues of it. Finerenone is non-steroidal selective MRA, with promising results in improving the deterioration of renal functions in CKD with DM, reducing albuminuria with less hyperkalemia along with improvement in cardiovascular outcomes by reducing heart failure events.

**Conclusion:**

Mineralocorticoid receptor antagonists have a proven role in preventing the adverse effects of RAAS pathway on heart, kidneys and blood vessels. Non-selective steroidal MRAs have potent action but by virtue of their non-selectivity associated with adverse effects like gynecomastia, hirsutism along with hyperkalemia Finerenone is novel non-steroidal & highly selective MRA, with promising results in halting the deterioration of renal functions in CKD with DM, reducing albuminuria, improvement in cardiovascular outcomes by reducing heart failure events albeit with less hyperkalemia. More randomized studies in dedicated HF patients are ongoing with Finerenone to prove it is worth in this sector with huge unmet need despite GDMT. Finerenone alleviates the risk of adverse renal and cardiac outcomes in patients with diabetes and CKD despite baseline medical therapy.

## Background

From the cortex of the adrenal glands a hormone of mineralocorticoid family is produced known as aldosterone. The main action of aldosterone is to promote reabsorption of Na^+^ along with water parallelly inciting K^+^ excretion, simultaneously adding pH balances at various cellular epithelium locations, including the distal convoluted tubules, collecting duct, salivary glands, and the gut. Aldosterone has demonstrable effects by acting on ENaC channels, Na^+^-K^+^ exchanger pumps, H^+^ ATPases and HCO^3^-Cl^−^ antiporters and exerts various non-epithelial functions specifically in cardiovascular disease processes, as in cell growth, inflammation and fibrosis [1, 2]. Its blockade by oral mineralocorticoids receptor antagonist (MRA) can impact upon altered structural and functional relationships in the heart, kidney, and various vascular beds [2–6].

The first MRA-Spironolactone was first developed in the 1950s, primarily as a potassium (K^+^) sparing diuretic but now considering their role in protection from non-epithelial side effects of aldosterone, they are being extensively utilized in heart failure (HF), refractory hypertension, and diverse nephropathies namely, diabetic kidney disease and proteinuria. Spironolactone is a non-selective steroidal aldosterone antagonist it is structurally similar to progesterone and binds to progesterone, androgen and mineralocorticoid receptors. Eplerenone is another steroidal MRA with selective inhibition and lacking the hormonal side effects of spironolactone enumerated earlier.

Both the old MRAs have been extensively used in treating hypertension, heart failure, proteinuria but have been associated with hyperkalemia, and deteriorating renal dysfunction [7].

To the existing family of MRAs another novel agent has been recently added—Finerenone (originally BAY 94-8862), a new MRA with selective action, having a dose-dependent benefit over older MRAs, decreasing rates of albuminuria and levels of BNP / NT-Pro BNP without causing a significant increase in serum potassium levels. The clinical use of Finerenone has begun in cases with nephropathy due to diabetes and results from studies in heart failure are keenly awaited [8]. These patients will be gaining from decrease in albumin excretion, decreasing the natriuretic peptide levels, and with negligible effects on eGFR.

In this review, we analayze Finerenone’s properties as an advanced MRA with a special action profile in CKD with type 2 diabetes mellitus and HF and also the differences with conventional old steroidal MRAs will be highlighted.

Further ahead will be discussing the role of RAAS inhibition in Cardiovascular diseases, the current scenarios with existing MRAs, pharmacological properties of Finerenone along with its various trials in HF and DKD, clinical implication and the future role of this newly developed versatile MRA.

## Main text

### Role of renin angiotensin–aldosterone system inhibition in cardiovascular diseases

The renin–angiotensin–aldosterone system (RAAS) is a powerful hormone regulatory mechanism serving multiple functions as a key determinant of tissue perfusion pressure and cellular homeostasis [9, 10].

**Fig. 1:**
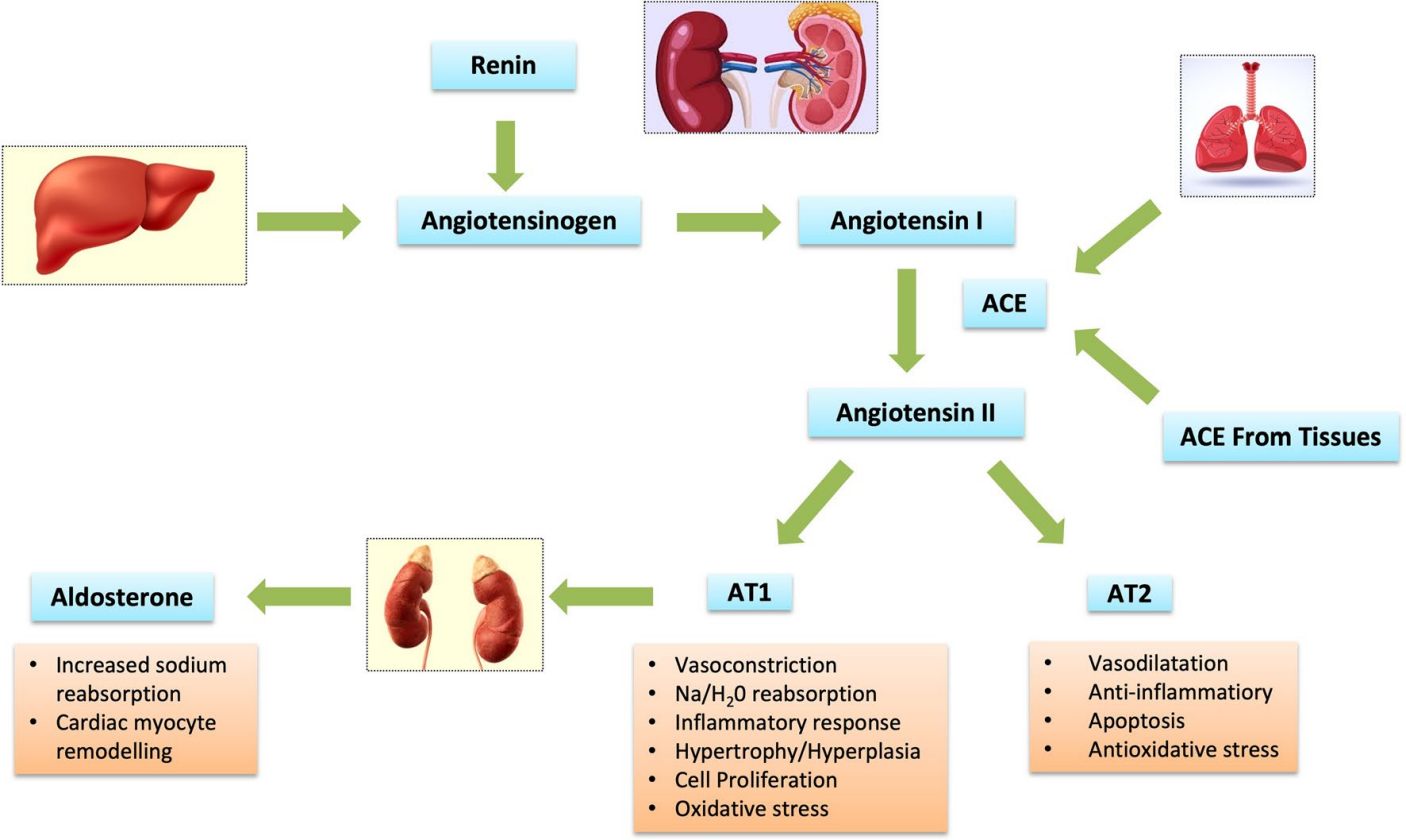
Pathophysiological effects of Renin Angiotensin and aldosterone system [1–3]. *ACE* Angiotensin converting enzyme, *AT* Angiotensin receptor

The mechanism for RAS activation in HF starts from renal hypoperfusion and consequently decreased Na+ load reaching the macula densa in the DCT leading to increased stimulation from the sympathetic system of the kidneys activating the juxtaglomerular apparatus causing the renin release. Renin cleaves the circulating liver synthesized angiotensinogen to form biologically inactive decapeptide Angiotensin I. Further action of the ACE enzyme leads to the formation of an octapeptide Angiotensin II. Most ACE is derived from tissues and the remaining is found in the non-membrane bound form in the heart and vessel wall interstitium. Kallikrein and cathepsin G enzymes can also produce Angiotensin II using renin independent pathways. Angiotensin II can undergo further degradation to generate three other biologically active fragments Angiotensin III, IV and 1–7.This usually occurs by ACE 2 partially homologous to ACE. This ACE 2 has been identified recently to be the receptor for COVID virus. Angiotensin II exerts its effects by binding to GPCRs, namely AT1 and AT2 receptors. The AT1 receptor is ubiquitous in the blood vessels; while, AT2 receptor is predominantly present in the human myocardium. The actions derived from these receptors have been described in Fig. 1. Increased levels of Angiotensin II is maladaptive, leading to fibrosis in the heart, kidney and other organs, it further worsens the neurohumoral activation, stimulating the zona glomerulosa to the adrenals causing the release of aldosterone hormone. This entire process of RAAS activation has been described in Fig. 1.

**Fig. 2:**
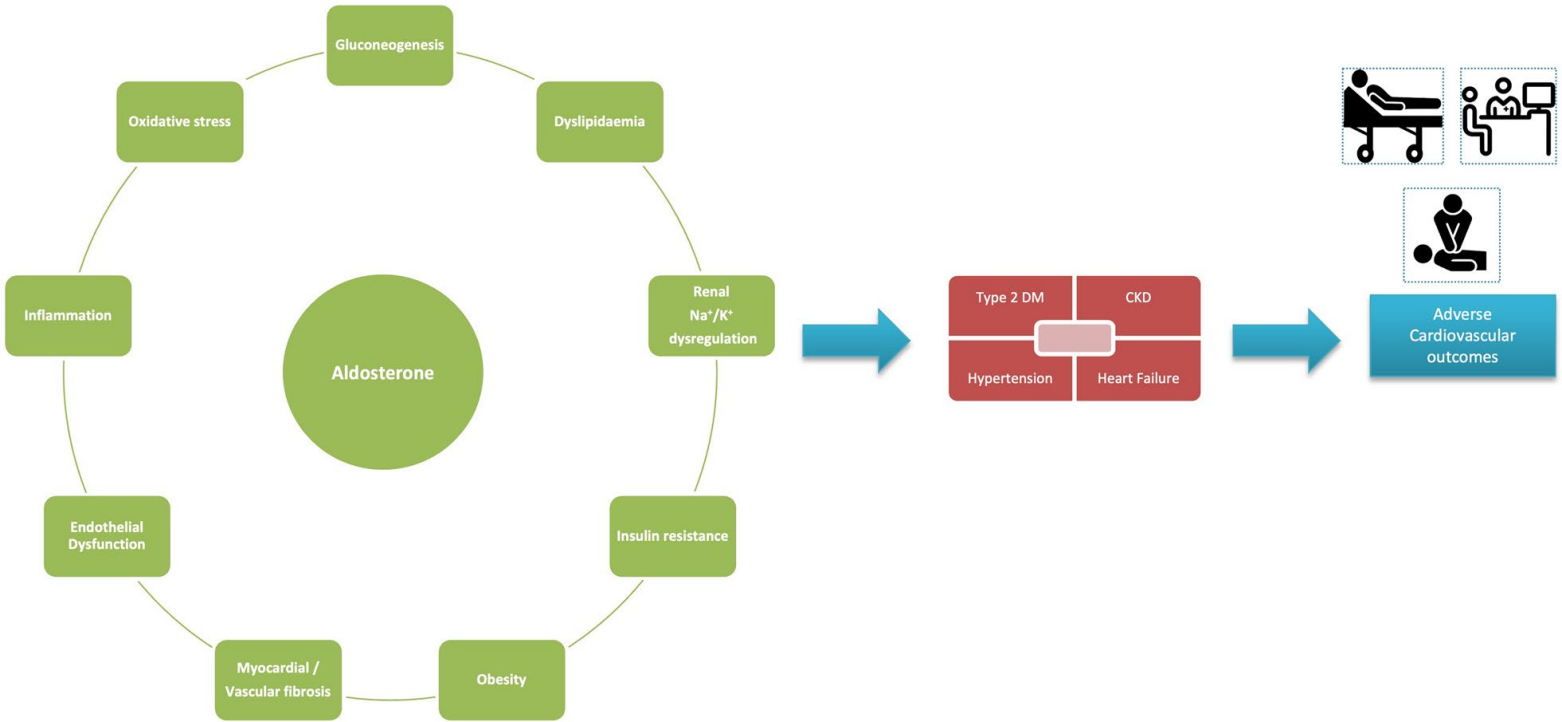
The detrimental Effect of Aldosterone on various organ systems [4–7]. The left panel depicts the various pathophysiological effects of aldosterone which leads to one or more of disease phenotypes in the patient (the central panel). The ultimate culmination is the occurrence of adverse cardiovascular outcomes which may be in the form of clinical sign/symptoms, hospitalizations and even mortality. *CKD* Chronic kidney disease, *DM* Diabetes mellitus

Aldosterone like Angiotensin II, exerts short term support to the circulation by increasing the sodium reabsorption exchanging it with K^+^ in the Distal tubule of kidney. Aldosterone effects are mediated by either genomic (slow) or non-genomic (rapid) mechanisms and has various detrimental effects on the heart, vessels and the kidneys, provoking fibrosis and hypertrophy within the vasculature and the myocardium further leading to reduce compliance of the vessel wall and hypertrophy of the ventricular mass. Endothelial dysfunction, baroreceptor dysfunction, reduced norepinephrine uptake leading to deterioration of heart failure. Aldosterone increases the oxidative stress and inflammation in the target tissues. In vascular smooth muscle cells (VSMCs), aldosterone induces a proliferative response through increased expression of p53-binding protein [9]. The cycle of activity and effects of aldosterone is elucidated in Fig. 2.

Complementing the understanding the pivotal pathological contribution of the RAAS system across the cardio-reno-metabolic continuum, the various drugs acting on the steps of RAAS system have proven to be cardiovascular and reno-protective. First in the list are ACE inhibitors that block the conversion of angiotensin I to angiotensin II leading to enhanced natriuresis, decreased blood pressure and prevents remodeling of smooth muscle and cardiac myocytes. Angiotensin Receptor Blockers form the second line of defense by acting on the AT1 receptors and nullifying the detrimental effects caused by its stimulation.

### Mineralocorticoid receptor antagonists (MRAs)

Aldosterone causes various detrimental effects on the cardiovascular and renal system that has already been described, which all starts from increased salt and water retention and increased excretion of K+ and H+ [11] (Fig. 2).

Spironolactone and Eplerenone are 1st and 2nd generation aldosterone antagonists, respectively, currently in wide use, acting on distal nephron inhibiting Na+/K+ at the site of aldosterone action [12].

Spironolactone is a non-selective antagonist of aldosterone that can bind to androgen and progesterone receptors as well. It is a steroidal 17α-spironolactone, better conceptualized as a derivative of progesterone, which is responsible for its potent anti-androgenic activity [13]. Mostly used in the treatment of a heart failure, its other uses include treatment of edema in nephrotic syndrome, ascites in liver diseases, essential hypertension, secondary hyperaldosteronism, and low potassium levels as in Barters’ syndrome [14, 15]. It is a weak diuretic as it acts on the collecting tubules. Because of its anti-androgenic activity it is also used in various dermatological conditions like acne, seborrhea and hirsutism.

The seminal trial for spironolactone—RALES demonstrated that spironolactone reduced overall mortality, mortality and hospitalization due to cardiac causes and the combined end point of death among patients with severely decreased left ventricular function resulting in advanced heart failure and who were receiving standard treatment including an angiotensin converting enzyme inhibitor [16]. Spironolactone in doses of 12.5–25 mg daily was pharmacologically effective in blocking the aldosterone receptors and decreasing atrial natriuretic peptide concentrations but serious hyperkalemia occurred most frequently with daily doses of 50 mg or greater [17]. Eight percent of the patients stopped the drug in the spironolactone group because of adverse events primarily due to gynecomastia. The more recent ATHENA-HF trial compared high dose spironolactone to spironolactone at low doses in acute HF with elevated natriuretic peptides. Although, the higher doses was well tolerated but there was not much difference in primary endpoint—NT-pro-BNP change at 96 h [18]. The side effect is a great concern among the treating physicians and led to the evolution of a selective MRA with non-steroidal properties—Eplerenone. It was derived from spironolactone by the introduction of a 9α,11α-epoxy bridge and by substitution of the 17α-thoacetyl group of spironolactone with a carbomethoxy group [19]. Eplerenone is up to 40-fold less potent than spironolactone at the MR, but it displays much greater selectivity for the MR over other steroid hormone receptors, which reduces the risk of sex hormone-related adverse effects, and is less potent in vivo as an anti-mineralocorticoid agent However, in contrast to spironolactone, eplerenone has little affinity for the androgen, progesterone, and glucocorticoid receptors [20]. Eplerenone differs from spironolactone in it’s extensive metabolism, with a short half-life , inactive metabolites and more consistent non genomic properties [21]. Eplerenone seems to be about 50 to 75% as potent as spironolactone for anti-mineralocorticoid activity. Hence, 25 mg/day spironolactone may be equivalent to approximately 50 mg/day eplerenone [21]. The Eplerenone Post-myocardial infarction Heart failure Study (EPHESUS) determined the effect of the MR antagonist eplerenone on the disease and associated deaths in cases with MI [22]. During a mean 16-month follow-up, patients with acute MI having complications like left ventricle failure assigned to eplerenone and associated optimal medical therapy, demonstrated significantly reduced morbidity and mortality. In the EMPHASIS-HF study, which had patients of NYHA Class II with left ventricular systolic dysfunction, showed reduced rate of hospitalization and death when treated with eplerenone [23].

The Trial of Aldosterone Antagonist Therapy in patients with heart failure with preserved ejection fraction (TOPCAT) has addressed the potential benefit of spironolactone on 3445 patients with symptomatic HF and HF with preserved ejection fraction [24, 25]. The results have been disappointing because only hospitalization was reduced by aldosterone antagonism; whereas, other end points such as the primary composite outcome of mortality from cardiac causes, aborted cardiac arrest, or hospitalization for HF did not differ statistically with the placebo arm. Based on these findings of the trial the 2022 AHA HF guidelines have given a Class 2b recommendation to MRA in HFpEF.

### Finerenone: the different one !

Finerenone or BAY94-8862 is a MRA with high selectivity and without a steroidal structure [26]. Finerenone was granted FDA approval on 9 July 2021, followed by the EMA approval on 11 March 2022. It is sold by the brand name Kerandia in 10 or 20 mg tablets. Finerenone has lesser affinity the receptors of steroid hormone compared to existing MRAs and hence is less prone to hormone receptors related off target effects than contemporary aldosterone antagonists such as spironolactone and eplerenone [27], while it continues to behave like a potassium sparing diuretic [28]. Finerenone is a complete MRA with a mixed mechanism of antagonism involving impairment of several steps in the MR signaling pathway [28].

Antagonism at the mineralocorticoid receptors is the potential reason for the CV benefits of these medications; while, antagonism of the secondary receptors causes undesired side effects. The affinity for estrogen receptors is negligible for all the three drugs. Table 1 depicts the inhibitory (blocking) concentrations (IC50, unit: nM) of the three anti-mineralocorticoids. In the lower the values, the stronger is the receptor inhibition.

**Table 1:** Comparison of inhibitory (blocking) concentrations (IC50, unit: nM) of the three available anti-mineralocorticoid agents

	Spironolactone	Eplerenone	Finerenone
Mineralocorticoid receptor	24	990	18
Glucocorticoid receptor	2400	22,000	> 10,000
Androgen receptor	77	21,200	> 10,000
Progesterone receptor	740	31,200	> 10,000

Mineralocorticoid receptor inhibition is responsible for the potential cardiovascular benefits of the drugs; whereas, inhibition of the other receptors potentially leads to side effects. The lower values indicate stronger inhibition [29]

In patients with mineralocorticoid receptors (MR) harboring the S810L genetic mutations treatment with conventional MRA can be a challenge. The S810L mutations refer to specific genetic alterations in which the serine (S) at position 810 of a protein is replaced by leucine. This gain of function mutation leads to conformation change in mineralocorticoid receptor and early onset hypertension as many antagonists are now paradoxically able to activate the MR [29]. While spironolactone and eplerenone act as agonists, Finerenone is known to exert its antagonist properties over there also [28, 30].

Finerenone is completely absorbed following oral administration with an absolute bioavailability of 43.5% as it gets metabolized passing the gut wall and the liver, C max is achieved between 0.5 to 1.25 h after dosing. Plasma protein binding is 92% primarily to albumin. The terminal half-life of Finerenone is about 2–3 h, and the systemic blood clearance is about 25 L/h. Finerenone is primarily metabolized by CYP3A4 (90%) and to a lesser extent by CYP2C8 (10%) to inactive metabolites. About 80% of the administered dose is excreted in urine and approximately 20% in feces (0.2% as unchanged) [31]. There are no clinically significant effects of age (18–79 years), sex, race/ethnicity (White, Asian, Black, and Hispanic), or weight (58 to 121 kg) on the pharmacokinetics of Finerenone.

There was no clinically significant difference in Finerenone pharmacokinetics when used concomitantly with gemfibrozil (strong CYP2C8 inhibitor), omeprazole (proton pump inhibitor), or an aluminum hydroxide and magnesium hydroxide antacid. There were no clinically significant pharmacokinetic differences for either Finerenone or concomitant digoxin (P-gp substrate) or warfarin (CYP2C9 substrate) [31].

It was seen in the various trials that at a dose 4 times the maximum approved recommended dose, Finerenone does not prolong the QT interval to any clinically relevant extent.

According to the data from various trials, Finerenone reduced the chances of persistent estimated Glomerular Filtration Rate reduction, progression to ESRD, CV deaths, MI (non-fatal), and hospital admissions for HF in cases with CKD due to T2DM [32].

**Fig. 3:**
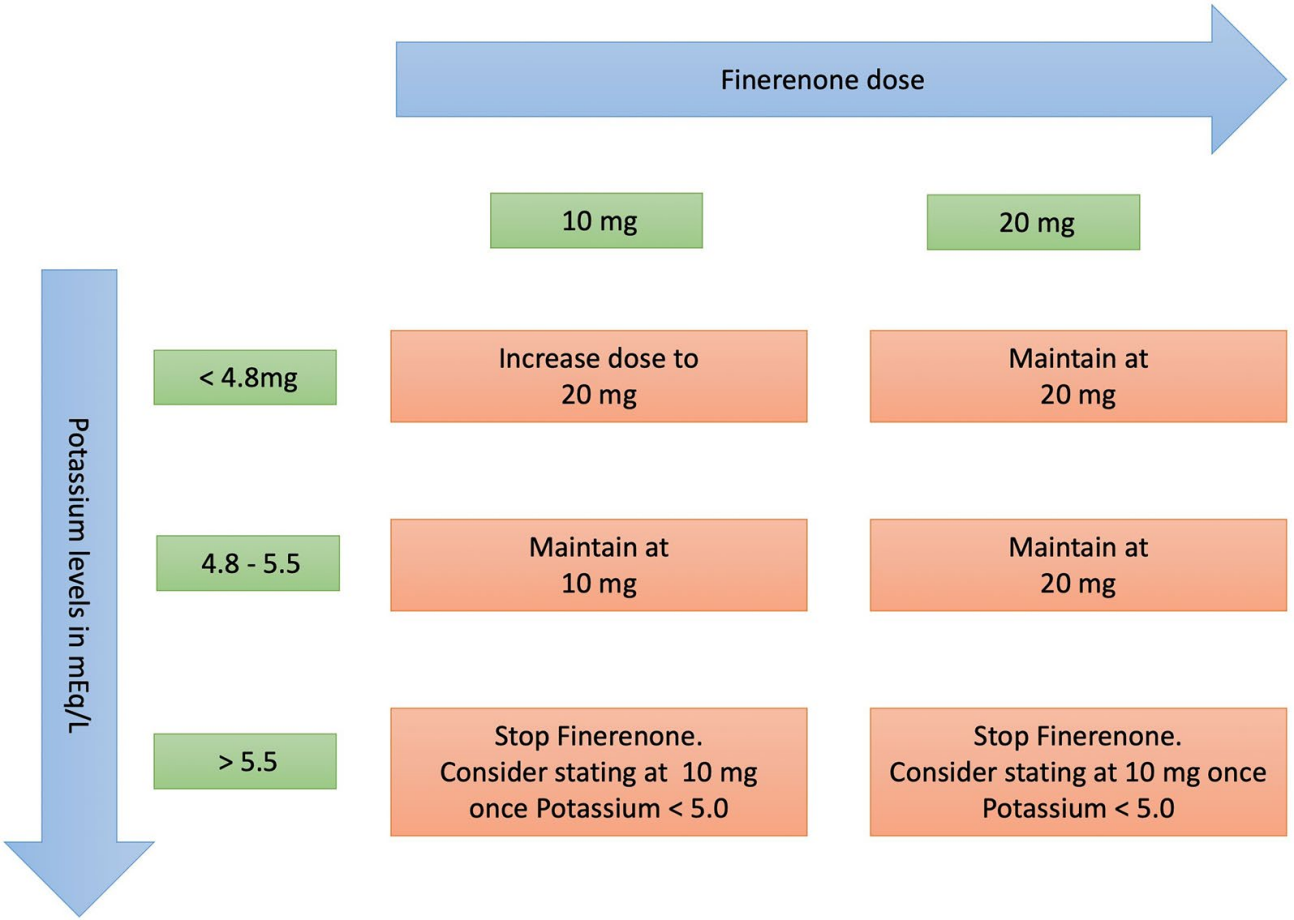
Suggested dose adjustment of Finerenone based on current (on-treatment) serum potassium concentration.[The figure has one error - the uppermost left orange box should be " Increase dose to 20 mg" in place of " Increase dose to 10 mg" ]

It is recommended to measure the eGFR and Serum Potassium levels prior to the initiation of Finerenone. The recommended starting doses for Finerenone according to the baseline eGFR levels are provided in Table 2. If eGFR has decreased by more than 30% compared to previous measurement, maintain a 10 mg dose. Figure 3 describes the dose adjustment of Finerenone according to serum K+ levels.

**Table 2:** Impact of baseline renal function (estimated GFR) on initial dosing of Finerenone in clinical practice

eGFR (in mL/min/1.73m^2^)	Starting dose
≥ 60 mL/min/1.73 m^2^	20 mg OD
≥ 25 to < 60 mL/min/1.73 m^2^	10 mg OD
< 25 mL/min/1.73 m^2^	Do not start

The physiochemical properties of non-steroidal MRA provide potential advantages compared with steroidal MRAs with respect to organ protective effects and a reduced risk of severe hyperkalemia. Hence, Finerenone has got better safety profile compared to the pre-existing MRAs in patients of heart failure and kidney diseases [31, 32].

Finerenone has the ability to provide cardiorenal benefit above the current standard-of-care treatment in high-risk patients with reduced renal function, and has also got a lower risk of hyperkalemia [33]. A study conducted in rats with neovascular retinopathy proved that Finerenone reduced injury to the blood vessels and up regulated T cells [34].

### Randomized control trials of finerenone and their implications

Finerenone has been extensively studied in various randomized trials, where its role as a MRA has been proven to be helpful in diabetic kidney diseases, attenuating the decline in the eGFR and reduction in CV events in such patients. Further ahead the results of the various trials in heart failure and CKD patients will be described, including the head to head comparison with existent MRAs.

ARTS-HF—This trial randomized around 1066 patients of with HF with reduced EF (with additional DM or CKD) to Finerenone versus eplerenone [33, 35]. Subjects were administered single daily dose of Finerenone, initiated at varying doses of 2.5–15 mg on the basis of body weight and dose was further doubled by day 30 (maximum dose of 20 mg) while in the other arm eplerenone was given 25 mg every alternate day, increased to a single daily dose at the end of the month and then to the double dose at the end of 2 months which was continued for 3 months. The percentage of subjects showing a decline in NT-pro BNP of > 30% after of 3 months was the primary end point. The secondary outcome was a combined endpoint of mortality from any cause, hospital admission due to cardiac issues, or worsening heart failure with need the for emergency care visit until Day 90. The ARTS-HF was the first study that was designed to compare the safety profile along with the effectiveness of five different treatment protocols with once-daily doses of Finerenone with another MRA (Eplerenone) in patients with concomitant T2DM and/or CKD, with chronic advanced HFrEF. At study completion, the decline in levels of biomarkers was no different among the two groups. NT-pro-BNP fall (> 30%) was observed in 37.2% in eplerenone group; while, it was 30.9–34.2% based on various doses in Finerenone group (*P* = 0.42–0.88). The composite of clinical end points was numerically lower in all Finerenone groups except for 2.5 mg baseline dose group.

The study also established that 10–20 mg is the most suitable dose for further testing in studies using Finerenone [35]. ARTS-HF Japan was also a small RCT comparing eplerenone with Finerenone that showed similar findings like ARTS-HF with similar risk of hyperkalemia particularly in Japanese patients [36].

In the pivotal FIGARO-DKD trial, there was significant improvement in cardiovascular outcomes with the drug compared to placebo. The study enrolled patients with type 2 DM with CKD stage 2 to stage 4 with moderately elevated albuminuria or stage 1 or 2 CKD with severely elevated albuminuria [37]. The improvement in CV outcomes seen in this study was predominantly as a result of decrease in the incidence of hospitalizations for HF (HHF) by Finerenone in patients with deranged renal functions and diabetes. At 3.4 years, the hazard ratio for HHF was 0.71 compared to placebo (95% CI 0.56–0.90) [38].

In another double blinded RCT FIDELIO-DKD, Finerenone was compared to placebo in cases with chronic renal failure and T2DM. The use of Finerenone resulted in reduced risk of renal failure progression and adverse cardiac events compared to that of a placebo [39, 40].

These two complimentary studies—FIGARO and FIDELIO examined cardiac and renal outcomes respectively, across the spectrum of CKD with type 2 DM. The purpose of the FIDELITY study was to perform a patient-level pre-specified pooled effectiveness and safety analysis of Finerenone (n > 13,000) across a broad range of chronic kidney disease. It was expected to provide a better estimation of efficacy and safety profile of the drug in comparison with a placebo. The analysis revealed that Finerenone reduced the risk of clinically important cardiac and renal outcomes vs. placebo across the various groups of CKD patients with DM [41]. Around 46 patients would need to be treated with Finerenone to prevent one cardiovascular outcome based on the absolute risk difference of 2.2% between groups.

In patients with T2DM, risks of cardiovascular mortality and heart failure (HF) increased with decreasing kidney function (based on eGFR) and increasing albuminuria (based on urine albumin-to-creatinine ratio [UACR]).In patients with high urine albumin creatine ratio, adding Finerenone reduced this ratio by reducing albuminuria in diabetic nephropathy patients [42]. Hence, Finerenone use favored better cardiac and renal outcomes in patients with chronic renal failure and diabetes in the FIDELITY (Finerenone in CKD and Type 2 Diabetes: Combined FIDELIO-DKD and FIGARO-DKD Trial Programme Analysis). Compared with placebo, Finerenone improved HF-related outcomes in patients with chronic renal failure and T2DM, with consistent benefits across eGFR and UACR sub-groups [41, 43]. There was no interaction of presence of left ventricular hypertrophy at baseline and cardiorenal benefits with Finerenone in the FIDELITY pooled analysis [44].

Two large scale meta-analyses estimating the effectiveness and any adverse effect profile of Finerenone in diabetic with renal failure in Chinese subjects, concluded that Finerenone plays a vital anti-proteinuric actions in subjects with CKD, and attenuated the adverse cardiac outcomes in these patients. It reduces the deterioration of renal functions specially in cases with moderate kidney disease on long term use, although the chances of hyperkalemia was more in the novel MRA group compared to placebo. But, there was no difference the chances of overall adverse events [45, 46].

A separate sub-study of FIDELITY analysis evaluated the impact of SGLT 2 inhibitors use on the benefits of Finerenone therapy. The conclusion was that UACR ratio improvement was observed in patients with chronic renal failure and diabetes mellitus independent of SGLT-2 inhibitors utilization at the baseline, and the positive effects on cardiorenal outcomes appeared to be present both groups whether SGLT2ihibitors were used or not [47]. Another subgroup analysis of FIDELITY strengthens the hypothesis that the renal and cardiovascular advantages are present with Finerenone irrespective whether being treated or not by a GLP 1 receptor antagonist [48].

In a FIDELITY pooled secondary analysis, anti-inflammatory effects of Finerenone were evaluated in chronically ill CKD patients with diabetes who had a greater risk of developing pneumonia and even greater risk of COVID-19 associated complications. It was concluded that Finerenone also protected against pneumonia and COVID-19 related adverse events [49].

### Finerenone as a new drug for HF: a critical analysis

Cardiovascular mortality and hospitalization due to heart failure in patients with DM with CKD is directly correlates with increasing albumin excretion and falling eGFR. Finerenone, a non-steroidal & selective MRA proved to be helpful in improving the cardiovascular outcomes in such patients as elucidated by FIDELIO-DKD, FIGARO-DKD trials and FIDLEITY pooled analysis. Finerenone greatly decreased the chances of 1st Hospitalization for Heart Failure (HR: 0.78 [95% CI 0.66–0.92]; *P* = 0.003), mortality (HR: 0.83 [95% CI 0.74–0.93]; *P* = 0.002), and cardiovascular death or recurrent Hospitalization for HF (HR: 0.82 [95% CI 0.72–0.95]; *P* = 0.006) in comparison with placebo. As established by the ARTS-DN study, Finerenone in addition with ACE inhibitors reduced albuminuria without causing additional adverse effects and causing any elevations on serum potassium levels [42]. Many studies have established the role of Finerenone in halting the progression of DKD and its predecessor albuminuria by reducing the decline in UACR [50, 51]. This protective effect also improves the cardiac function in such patients have the tendency to land up into decompensated heart failure due to volume overload. Finerenone has similar potency like spironolactone. As Finerenone has minimal renal excretion, it is best suited MRA for use in patients with reduced renal functions. Finerenone use had a lower overall of negative event rates when a comparison was done with spironolactone in patients with chronic HFrEF plus CKD and with almost equal incidence to eplerenone in cases with a chronic HFrEEF plus CKD and/or diabetes mellitus [51]. Gynecomastia was not reported with Finerenone at all!

The dual blockade with the use of Finerenone provides easy and more complete amelioration of the of RAAS pathway. The cardiovascular benefits were seen in patients with or without pre-existing cardiovascular diseases [52]. In a subset of patients with CKD and Diabetes, Finerenone has also been shown to reduce the risk of developing new onset Atrial flutter or fibrillation [53].

The ARTS-HF trial primarily focused on patients with worsening HF with type 2 DM with or without renal dysfunction, comparing eplerenone versus Finerenone. The reduction of NT-pro BNP was equal in both groups. In totality, cases in the Finerenone 10 mg group with dose escalation 20 mg had the maximum decline in the composite results including all-cause mortality, hospital admissions due to cardiovascular issues, or landing to the hospital in emergency department, compared with patients in the Eplerenone (HR for all-cause death: 0.56; 95% CI 0.35, 0.90). This dosing protocol appears to achieve the equipoise between an efficacy and safety for future trials. Thus, effectively in a dose of 10 mg and up titrated to 20 mg over 3 months Finerenone could be an equivalent to eplerenone in treating patient of long standing HFrEF with equal safety profile. The lower doses of Finerenone didn’t prove to be much helpful. Further, research is needed to explore subgroup of patients who might benefit from doses other than standard ones (10 mg/20 mg) of Finerenone as a cardio protective agent. To sum up, Finerenone does prove to be effective in improving cardiovascular outcomes in patients having CKD with Diabetes, with potency as good as spironolactone and safety equal to eplerenone.

### The future of finerenone

The most recent and well formulated 3rd generation MRA in vogue is Finerenone. The classic aldosterone antagonists have been in use for reduction of hospital admissions and death in long standing HF but the use gets restricted in clinical practice due to the unavoidable chances of increase in potassium, reduction in kidney functions, gynecomastia in males, and hormonal changes in women. Finerenone is more selective than spironolactone toward mineralocorticoid receptor (MR), and has very little or no affinity for androgen, glucocorticoid, and progesterone receptors, similar to eplerenone. It’s receptor selectivity is better than spironolactone while comparing with eplerenone it has greater affinity. Finerenone achieves a greater balance between efficacy and renal side effects, especially in populations prone to hyperkalemia such as patients with CKD or DM [32]. In contrast to existing MRAs having a higher tendency to focus on the kidneys rather than the heart, Finerenone exerts similar emphasis on heart and kidney tissues [54]. Recent analysis pertaining to the FIDELITY pooled data have also outlined an 18% reduction in all-cause mortality and cardiovascular mortality (on treatment analysis) with Finerenone; while, there was 25% lowering of sudden cardiac death also (intention to treat analysis) [55].

Moreover, the ongoing trial FINEARTS-HF is studying the role of Finerenone therapy in HFpEF with EF > 40% and evaluating the impact of the drug on morbidity and mortality in this population [56]. A pioneer study has revealed that Finerenone improves diastolic dysfunction and cardiac perfusion in preclinical heart failure with preserved EF model in rats and these cardiac benefits were largely independent of renal benefits. This seconds the postulations of the seminal FINEARTS-HF study [57]. This effect can be a game changer in the treatment of HFpEF [58]. Another phase III study, the FINALITY-HF (NCT06033950) is utilizing Finerenone in HFrEF patients who are unable to tolerate the conventional steroidal MRA [59]. It is a clinical end point driven study with time to first HF event or CV death as the primary goal. The REDEFINE-HF study looking at the role of the drug in HFrEF & HFmrEF patients who have been recently hospitalized with HF. The COMBINATION-HF aims to evaluate concomitant Finerenone & SGLT-2 inhibitor following an episode of acute HF.

It has been proved by various trials now that Finerenone reduces cardiac and renal adverse events in diabetic patients regardless of ASCVD, halting the disease progression on one hand improving the outcomes on the other. These vital benefits seen in the reno-cardio-diabetic continuum with the drug pertains in the direction of a holistic approach and will be certainly helpful in long term prognosis of diabetic patients. The potential arenas for Finerenone in treating atrial fibrillation, pulmonary hypertension, retinopathy and primary aldosteronism is under evaluation [60].

Non-Steroidal MRA are a new class of agents with potential benefits in the spectrum of Diabetes, CKD and heart failure with preserved efficacy and diminished side effects compared to conventional MRA. While Finerenone is the pro-type agent in the class, another new non-steroidal MRA Esaxerenone is also in pipeline [61].

## Conclusion

Mineralocorticoid receptor antagonists have a proven role in preventing the adverse effects of RAAS pathway on heart, kidneys and blood vessels [8]. Non-selective steroidal MRAs like spironolactone have potent action but by virtue of their non-selectivity associated with adverse effects like gynecomastia, hirsutism along with hyperkalemia; whereas, eplerenone has good potency with less adverse effects but hyperkalemia is still remains an area of challenge especially in patients with deranged renal functions like DKD/CKD. Finerenone is novel non-steroidal & highly selective MRA, with promising results in halting the deterioration of renal functions in CKD with DM, reducing albuminuria, improvement in cardiovascular outcomes by reducing heart failure events albeit with less hyperkalemia [62]. In Patients with chronic HF, it did not prove to be statistically superior to Eplerenone in reducing biomarkers but clinical exploratory endpoints were numerically lower with Finerenone. More randomized studies in dedicated HF patients are ongoing with Finerenone to prove it’s worth in this sector with huge unmet need despite GDMT. Addition of Finerenone will be benefitting patients with diabetes and CKD who are already on GDMT by alleviating the risk of adverse renal and cardiac outcomes [59].

## Data Availability

The data for review has been extracted from articles already published in the prestigious journals and have been acknowledged in the references.

## Abbreviations

CKD: Chronic kidney disease
MRA: Mineralocorticoid receptor antagonist
DM: Diabetes mellitus
HF: Heart failure
ENaC: Epithelial sodium channel
BNP: Brain natriuretic peptide
NT-PRO BNP: N-terminal pro brain type natriuretic peptide
eGFR: Estimated glomerular filtration rate
RAAS: Renin angiotensin aldosterone system
DKD: Diabetic kidney disease
DCT: Distal convoluted tubule
ACE: Angiotensin converting enzyme
GPCRs: G protein coupled receptors
AT I & AT II: Angiotensin receptor type I and type II
NYHA: New York Heart Association
Nm: Nanomole
FDA: Food and Drug Administration
EMA: European Medicine Agency
IUPAC: International Union of Pure and Applied Chemistry
ESRD: End stage renal disease
CV: Cardiovascular
MI: Myocardial infarction
RCT: Randomized control trial
SGLT2: Sodium glucose co-transporters 2
GLP1: Glucagon like peptide 1
EF: Ejection fraction
HFrEF: Heart failure with reduced ejection fraction
ASCVD: Atherosclerotic cardiovascular disease
HFmrEF: Heart failure with mid-range ejection fraction
